# Investigation of the Effects of Rapid Thermal Annealing on the Electron Transport Mechanism in Nitrogen-Doped ZnO Thin Films Grown by RF Magnetron Sputtering

**DOI:** 10.3390/nano12010019

**Published:** 2021-12-22

**Authors:** Simeon Simeonov, Anna Szekeres, Dencho Spassov, Mihai Anastasescu, Ioana Stanculescu, Madalina Nicolescu, Elias Aperathitis, Mircea Modreanu, Mariuca Gartner

**Affiliations:** 1 Institute of Solid State Physics, Bulgarian Academy of Sciences, 72 Tsarigradsko Chaussee, 1784 Sofia, Bulgaria; simeon@issp.bas.bg (S.S.); d_spassov@abv.bg (D.S.); 2 Institute of Physical Chemistry “Ilie Murgulescu”, Romanian Academy, 202 Splaiul Independentei, 060021 Bucharest, Romania; manastasescu@icf.ro (M.A.); mgartner@icf.ro (M.G.); 3 Horia Hulubei National Institute of Research and Development for Physics and Nuclear Engineering, 30 Aleea Reactorului, 077125 Magurele, Romania; istanculescu@nipne.ro; 4 Department of Physical Chemistry, Faculty of Chemistry, University of Bucharest, 4-12 Regina Elisabeta Bd., 030018 Bucharest, Romania; 5 Microelectronics Research Group, Institute of Electronic Structure and Laser, Foundation for Research and Technology (FORTH-Hellas), P.O. Box 1385, 70013 Heraklion, Crete, Greece; eaper@physics.uoc.gr; 6 Tyndall National Institute-University College Cork, Lee Maltings, Dyke Parade, T12 R5CP Cork, Ireland; mircea.modreanu@tyndall.ie

**Keywords:** RF magnetron sputtering, ZnO:N thin films, Raman spectroscopy, photoluminescence spectroscopy, electrical characteristics, charge carrier transport properties

## Abstract

Nitrogen-doped ZnO (ZnO:N) thin films, deposited on Si(100) substrates by RF magnetron sputtering in a gas mixture of argon, oxygen, and nitrogen at different ratios followed by Rapid Thermal Annealing (RTA) at 400 °C and 550 °C, were studied in the present work. Raman and photoluminescence spectroscopic analyses showed that introduction of N into the ZnO matrix generated defects related to oxygen and zinc vacancies and interstitials. These defects were deep levels which contributed to the electron transport properties of the ZnO:N films, studied by analyzing the current–voltage characteristics of metal–insulator–semiconductor structures with ZnO:N films, measured at 298 and 77 K. At the appliedtechnological conditions of deposition and subsequent RTA at 400 °C n-type ZnO:N films were formed, while RTA at 550 °C transformed the n-ZnO:N films to p-ZnO:N ones. The charge transport in both types of ZnO:N films was carried out via deep levels in the ZnO energy gap. The density of the deep levels was in the order of 10^19^ cm^−3^. In the temperature range of 77–298 K, the electron transport mechanism in the ZnO:N films was predominantly intertrap tunneling, but thermally activated hopping also took place.

## 1. Introduction

Zinc oxide possesses remarkable optical and semiconductor properties, such as a direct wide gap around 3.3 eV at room temperature and a large exciton binding energy of about 60 meV [1]. Because of these properties, ZnO has huge prospects in applications such as optoelectronic devices [2], homojunction LEDs [3,4], solar cells [5], sensors [6,7], and other devices and structures. However, the use of ZnO films in these applications requires the ability to control the majority-carrier type and concentration. An asymmetry exists between n- and p-type doping of ZnO thin films. While it is possible to prepare stable n-type ZnO films even without the introduction of donor dopants during the ZnO film deposition, obtaining p-type ZnO film requires thermal activation of acceptor dopants incorporated into the ZnO film during its deposition [8]. In some cases, an additional problem is the subsequent transformation of the p-type ZnO films into n-type ZnO films [9]. This is why producing stable and qualitative p-type ZnO films is still a challenge facing the technologies of the above-listed devices and structures.

Much effort has been made to obtain p-type ZnO films by applying different advanced deposition methods [10,11,12,13,14] and introducing different elements into ZnO as additives [15]. Among the acceptor dopants, nitrogen is considered themost promising [9]. The nitrogen atom possesses three 2p valence electrons, while the oxygen atom has four 2p valence electrons. The size of the nitrogen atom and the energy of the N 2p valence electrons are closest to the corresponding values of the oxygen atom [16]. Therefore, one might expect the replacement of some O anions in the anion sublattice with N anions and the formation of shallow acceptor levels in the ZnO energy gap. However, to prepare a stable p-type ZnO film by N doping is rather difficult due to the low solubility of nitrogen, and thus the low concentration of holes in the films. Another obstacle to effective p-type doping via the replacement of O by N in the anion sublattice is the simultaneous generation of shallow and deep donor-type defects in the ZnO energy gap, such as oxygen vacancies in the O sublattice, V_O_, and zinc interstitials, Zn_i_ [17,18,19], which leads to self-compensation of N acceptors in ZnO [20]. Difficulties in the preparation of p-type ZnO films have provoked interest in the studies of ZnO heterojunctions, mainly ZnO–Si heterojunctions. The purpose of these investigations is to expand the applications of ZnO thin films in photoelectron devices and structures, especially in solar cell structures.

Zinc oxide is also the subject of our research. As a pathway for ZnO thin film deposition, we applied a radio frequency (RF) magnetron sputtering technique. This deposition technique combines the relative easiness of both nitrogen doping of the growing layers by the simple introduction of N_2_ into the process chamber with control of the deposition parameters (such as gas pressure, substrate temperature, scattering power, and deposition rate). The ability to precisely maintain these parameters during film deposition determines the reproducibility and efficiency of this method. Because of this, RF magnetron sputtering is one of the most widely used methods for thin-film deposition. In order to improve the properties of our ZnO:N films, we applied postdeposition rapid thermal annealing (RTA) in a nitrogen atmosphere at different temperatures. The choice of RTA was due to the fact that it is more technologically relevant than conventional thermal treatments that take much longer and are more highly energy consuming. Moreover, during the RTA process, nitrogen release from the ZnO:N layer is less probable. It has been shown that the RTA method is a particularly suitable method for improving the structural, optical, and electrical properties of ZnO films [21,22].

In order to use nitrogen-doped ZnO films in ZnO homo- and heterojunctions and/or as transparent conductive oxide films, a detailed characterization of their structural, optical, and electrical properties is required. It is particularly important to understand, and hence to tailor, the charge transfer processes in these materials. Accordingly, detailed structural studies including X-ray Diffraction (XRD), Transmission Electron Microscopy (TEM), Atomic Force Microscopy (AFM), and Scanning Electron Microscopy (SEM) were preliminarily conducted on the RF-sputtered N-doped ZnO films [23,24,25]. We established that RTA treatment in N_2_ at moderate temperatures essentially improves the crystalline state of the ZnO films, magnetron sputtered onto crystalline silicon [23], or fused silica [24] substrates, yielding a polycrystalline columnar structure with nanocrystallites of 9–13 nm, preferentially oriented in the (002) direction. In these studies, the ZnO films were deposited in a gas mixture of Ar:O_2_:N_2_ = 50:40:10 and at a total pressure of 0.66 Pa (5 m Torr). Further, we collected the same measurements as mentioned above on ZnO films deposited under increased amounts of N_2_ and a constant amount of Ar. It was established that variation of the Ar:O_2_:N_2_ mixture to 50:25:25 and 50:10:40 weakly affected the films’ crystallinity and surface morphology (the root-mean-square roughness values being lower than 5 nm), but influenced the optical properties [25], and hence alteration in the electrical properties is also expected.

The main purpose of the present research was to investigate the electrical properties of RF magnetron-sputtered nitrogen-doped ZnO films and to elucidate the charge transport mechanism in these films. There are few data in the literature concerning the charge carrier transport through N-doped ZnO films and, peculiarly, the charge transport parameters. For this purpose, we prepared Al–ZnO:N–Si–Al structures and their electrical properties were studied in detail. In addition, Raman and Photoluminescence (PL) measurements were carried out, aiming to establish the origin and nature of the deep levels associated with inherent defects in the polycrystalline ZnO:N films. This could shed more light on electron transitions and charge carrier transport involving N-induced defective centers. The final goal of the research was elucidation of the electron transport properties of zinc-oxide-based films, which would expand their application area in optoelectronics, for example as transparent ZnO:N n-p homojunctions.

## 2. Materials and Methods

### 2.1. Film Preparation

The nitrogen-doped ZnO thin films were deposited by RF magnetron sputtering employing Nordiko RFG-2500 equipment (Nordiko Technical Service Ltd., Havant, Hampshire, United Kingdom). Commercially available zinc nitride target (Testbourne Ltd., Basingstoke, Hampshire, UK), Zn:N = 1:1, purity 99.9%, 6 in diameter×0.25 in thick) was sputtered in a gas mixture of Ar, O_2_, and N_2_ onto p-type Si(100) substrates with specific resistivity over 100 Ω cm. In general, the substrates never occupied an area bigger than ~12 cm^2^ on the holder. The distance between the target and substrates was 11 cm.

The substrates were ultrasonically cleaned in acetone and isopropanol, rinsed in deionized water, and dried in flowing nitrogen gas. The native oxide on the Si surface was etched in HF solution before the substrates were introduced into the sputtering system. After reaching the base pressure of 1.33 × 10^−5^ Pa (10^−7^ m Torr) and prior to deposition, the target was presputtered for at least 15 min (Ar plasma, 0.66 Pa (5 m Torr), 100 W RF power) to remove any contaminants from the target surface and to enable equilibrium conditions to be reached. The RF power was kept at 100 W. The sputtering was carried out at a total pressure of 0.66 Pa (5 m Torr) in a gas mixture of Ar:O_2_:N_2_, keeping Ar constant at 50% and varying the ratio of O_2_ and N_2_ percentages: (i) O_2_:N_2_ = 40:10, (ii) O_2_:N_2_ = 10:40, and (iii) O_2_:N_2_ = 25:25. During deposition, no external heating was applied to the substrates. At the given technological conditions, the deposition rate of the ZnO films was 0.83 nm/min for the films made in the oxygen-rich plasma and 0.32 nm/min for the films made in oxygen-deficient plasma.

After deposition, and following our previous investigations concerning postdeposition annealing conditions as mentioned above [23,24,25], the ZnO:N samples were subjected to RTA at temperatures of 400 °C and 550 °C for 1 min in N_2_ atmosphere. The annealing temperature of 550 °C was not exceeded in order to avoid any possible release of nitrogen from the structure of the films [26].

The denotations of the ZnO:N–Si samples prepared under different technological conditions are presented in Table 1. In the table, we have included the thicknesses of the corresponding films, which were determined with an accuracy of ±0.2 nm from the spectral ellipsometric measurements. The scatter of the as-deposited films’ thickness resulted mainly from the low deposition rates and different sputtering durations. It should be pointed out that the thickness uniformity of the films deposited on given Si samples was ~96%, as established by the ellipsometric mapping [25].

The bandgap energy values of ZnO:N films deposited under the same technological condition as were used in the present work have been determined from the ellipsometric data analysis published elsewhere [25]. It was found that for the as-deposited ZnO:N films, the bandgap energy value was 3.25 ± 0.025 eV, while for the 400 °C and 550 °C RTA samples there was a slight reduction toward 3.20 ± 0.025 eV. These optical bandgap energy values are in good agreement with the literature reports [17,19,27,28]

### 2.2. Characterization Methods

Raman scattering spectra of the films were recorded on a complex configuration Bruker Vertex 70 FTIR/FT-Raman instrument (Bruker, Ettlingen, Germany). The spectrometer equipped with a RAM II module using a Nd:YAG laser (1064 nm) with variable power (1–500 mW) and LN2 cooled Ge detector. The Fourier-transform Raman spectra were registered between 200 and 600 cm^−1^ with 512 scans and 4 cm^−1^ spectral resolution, and a laser power of 1 mW.

The photoluminescence (PL) measurements were carried out on a Carry Eclipse (Agilent Technologies, Melbourne, Australia), fluorescence spectrometer, with the slits set at 20 nm. The photoluminescence at room temperature was excited by the 325 nm line from a Xe bulb lamp. The PL spectra were taken with a scan rate of 120 nm/min and spectral resolution of 0.5 nm.

The electrical measurements were conducted on metal–ZnO:N–silicon capacitors (further denoted as metal–insulator–semiconductor (MIS) structures) formed by vacuum thermal evaporation of Al dots with 1.96 × 10^−3^ cm^2^ area onto the ZnO top surface through a metal mask, while continuous Al film was evaporated as a contact to the silicon backside.

The electrical properties of the MIS structures were examined from the current–voltage (I–V) characteristics, measured in two ways. In the first way, the I–V characteristics were automatically measured at room temperature in a dark chamber using a Keithley 4200 Semiconductor Characterization System with a ramp rate of 0.1 V/s. In the second way, the I–V curves were measured point by point with a cycle sequence starting from 0 V toward negative or positive voltages, with maximal amplitude applied to the top Al–dot contact followed by a voltage reversal toward zero voltage. The first measurement cycle starting from zero up to maximal applied voltage, V_a_, is denoted further as the initial stage. The duration of each measurement cycle was approximately 20–25 min. This kind of I–V measurement was applied to samples 2.1 and 2.3 at temperatures of 298 and 77 K. In this way, the presence and behavior of deep levels in the ZnO:N films could be examined and their concentrations determined.

In addition, the impedance of the MIS structures with ZnO layers 2.1 and 2.3 was measured at room temperature with a Tesla BM-507 impedance meter (TESLA, Praha, Czechoslovakia) applying test voltage frequencies in the range of 0.5–500 kHz. Herein, we consider only the parallel conductance, G_m_, values, calculated from the measured |Z_m_| and φ_m_ quantities using the expression G_m_ = cos(φ_m_)/|Z_m_|.

## 3. Results and Discussion

The structural and morphological properties of the ZnO:N films (Table 1), as well as their chemical compositions, were reported in our previous papers [23,24,25]. XRD investigations revealed that all ZnO:N films were crystallized in a hexagonal ZnO wurtzite phase (JCPDS data card 36-1451), (002) oriented, and exhibited an improvement in crystallinity after RTA. There was no noticeable change in the XRD patterns induced by the variation of the nitrogen content in the sputtering reactor atmosphere during ZnO:N film deposition, but the values of the crystallite size, estimated using the Scherrer formula, increased with the RTA temperature and with N_2_ content in the sputtering atmosphere, from 14.4 nm (10% N_2_) to 15.7 nm (40% N_2_) for the ZnO:N films after RTA at 550 °C [25]. From a topographic point of view, all ZnO:N–Si samples (Table 1) showed a homogeneous distribution of small and rounded superficial grains, with an average diameter in the range of 40–70 nm, all surfaces being smooth, with a root-mean-square (RMS) roughness of lessthan 5 nm. From TEM investigations, it was found that the morphology of the ZnO:N films was columnar, with the grain column axis oriented nearly parallel with the hexagonal <001> axis of the ZnO structure. The columnar structures had variable diameters from 10 nm at the bottom to 50 nm at the top (for the films sputtered under 40% nitrogen), so that the surface had larger grains which were well faceted, as seen by AFM. The columns of the as-deposited films contained a lot of defects and pores which diminished after RTA. The presence of the nitrogen inside the ZnO:N films was not detected by XRD analysis due to its very small amount, but was evidenced in Energy-dispersive X-ray spectroscopy (EDX) observations conducted in a ratio of 1/6 (nitrogen/oxygen) for the films deposited on Si, regardless of the RTA treatment. However, the presence of nitrogen was clearly pointed out by X-ray photoelectron spectroscopy (XPS) in the RTA samples only as zinc nitride and diluted zinc oxynitride, as reported previously [23,24]. Nevertheless, the uniformity of thickness deposition, determined by ellipsometric mapping, was found to be around 96% for the ZnO:N films deposited on silicon [25].

### 3.1. Analysis of FT-Raman Spectra

**Figure 1:**
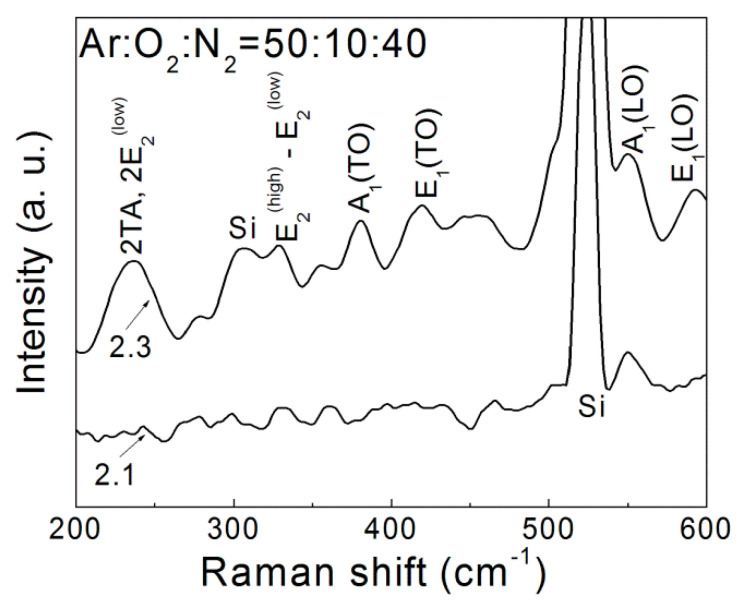
Representative Raman spectra of ZnO:N films, deposited in a gas mixture of Ar:O_2_:N_2_ = 50:10:40 (sample 2.1) and after RTA at 550 °C (sample 2.3).

In Figure 1, as a representative illustration, the Raman spectra of the ZnO:N film deposited in gas mixture of Ar:O_2_:N_2_ = 50:10:40, (sample 2.1) and RT annealed at 550 °C (sample 2.3) are given. The nonsymmetrical shape of the Raman peaks is due to the multiple contributions from the polycrystalline nature of the ZnO:N [25]. The vibrational bands of Si–Si bonds are also indicated, appearing as a strong peak at 523 cm^−1^ and as a weak and broad peak in the 303–308 cm^−1^ spectral range. The kind and position of the observed vibrational modes of ZnO:N films in dependence on the oxygen/nitrogen content and RTA temperature are summarized in Table 2.

Pure, crystalline ZnO has eight sets of optical phonon modes at the Γ point in the Brillouin zone, Γ_opt_, expressed as Γ_opt_ = A_1_+ E_1_ (IR, R) + 2E_2_ (R) + 2B_1_ [29]. The A_1_ and E_1_ modes are both Raman and infrared active and split into transverse optical (TO) and longitudinal optical (LO) phonon modes. The E_2_ modes are Raman active and nonpolar at low- (E_2_^(low)^) and high-frequency (E_2_^(high)^) phonon modes. The E_2_^(low)^ mode involves mainly Zn sublattice motion while E_2_^(high)^ is associated with the vibration of oxygen atoms [30]. There are also two B_1_ modes, B_1_^(low)^ and B_1_^(high)^, which are inactive Raman modes but can be activated by introducing defects or by doping with other elements [29,31,32].

The recorded FT-Raman spectra exhibited the state of molecular Zn–O bonds in the ZnO:N films under changing technological conditions. The increase of the peaks’ intensity after RTA treatment implies the increase of defects related to O and Zn vacancies and interstitials. The bands that appeared at 230–240 cm^−1^ and 330 cm^−1^ correspond to (2TA and 2E_2_^(low)^) and E_2_^(high)^-E_2_^(low)^, respectively, and are assigned to the contribution of second-order vibrations, two-phonon modes which become activated in thin films. The peak around 377 cm^−1^ was identified as the A_1_(TO) mode, which indicates the displacement of Zn^2+^ and O^2−^ ions parallel to the c-axis, near the center of the Brillouin zone (Γ point) [30,33,34]. In comparison to the pure crystalline ZnO, the position of the wide E_2_^(high)^ peak at 410–420 cm^−1^ was significantly shifted in the doped ZnO:N films, indicating that nitrogen doping mainly affected the oxygen bonds. The peak at 550 cm^−1^ was assigned to the inactive A_1_(LO) mode, activates by introducing defects or by doping with other elements [25,31,32,33]. The observed peak at 590 cm^−1^ was attributed to the E_1_(LO) mode that appeared due to oxygen vacancies V_O_, zinc interstitials Zn_i_, and free carriers [35,36] existing in the analyzed ZnO films. The Raman study proved that the incorporation of nitrogen into the films caused disordering of the ZnO lattice. Considering that the spectra were taken with a resolution of 4 cm^−1^, the close positions of the corresponding peaks indicated similar structure and chemical bonding. The exceptions were the peaks around 402–412 cm^−1^, confirming that the change in the oxygen/nitrogen ratio in the Ar:O_2_:N_2_ mixture predominantly affectedthe oxygen bonds in the ZnO lattice.

### 3.2. Photoluminescence Analysis

**Figure 2:**
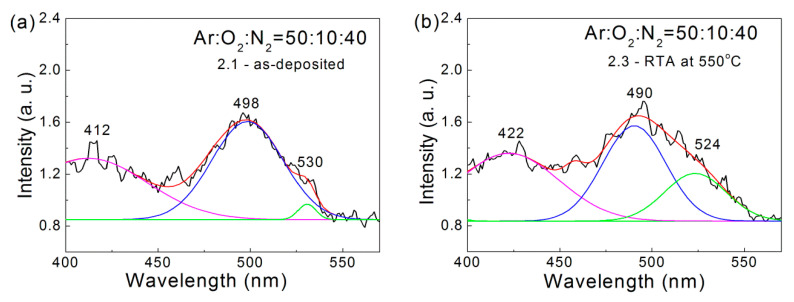
Representative PL spectra of the ZnO:N films deposited in a gas mixture of Ar:O_2_:N_2_ = 50:10:40:(**a**) as-deposited (sample 2.1) and (**b**) after RTA at 550 °C (sample 2.3).

In general, in the PL spectra of ZnO films, two emission bands can be observed: one is in the UV region within the 361–369 nm range and the second band is in the visible range of 450–550 nm [32,37]. As the excitation source (Xe bulb lamp) was not intense enough to excite the transitions associated with the optical band gap energy, the first characteristic band was not observed. In Figure 2, typical PL spectra in the spectral range of 400–560 nm are presented for a ZnO:N film deposited in gas mixture of Ar:O_2_:N_2_ = 50:10:40 (sample 2.1) and after RTA annealing at 550 °C (sample 2.3).

The representative PL spectra of the studied ZnO:N films were characterized by a weak band around 455–460 nm and more strong and broad bands around 412 nm and 500 nm. Due to the broad PL peaks, their locations were difficult to resolve accurately. For this reason, we tried to find the Gaussian components until the best fit was achieved. The dominant 500 nm band was deconvoluted into two Gaussian components as the peaks were situated around 490 and 524 nm.

The emission peak from the band-to-band recombinations was expected to appear around 382 nm, the wavelength corresponding to the bandgap energy (3.25 ± 0.025 eV) of our ZnO films. However, in the measured PL spectra, the emission peak was centered within the 410–420 nm region, and hence it originated most probably from defect states close to the ZnO bandgap edges. Previous research suggests that zinc interstitial defects, Zn_i_, creating shallow states underneath the conduction band edge, are a possible reason for the appearance of this PL peak [38]. The emission peak at ~490 nm (2.53 eV) was a typical blue green emission of ZnO, which may be attributed to a high density of point defects such as zinc vacancy, V_Zn_, oxygen vacancy, V_O_, and interstitial oxygen, O_i_, in the polycrystalline structure of ZnO [39]. The green emission peak at ~525 nm (2.36 eV) resulted from radiative recombination of holes with electrons at the singly ionized intrinsic oxygen vacancies [40,41]. These kinds of defects are deep levels, which contribute to the electron transport properties and were considered further at the data analysis of electrical measurements.

The positions of emission peaks obtained by Gaussian deconvolution of the PL spectra of ZnO:N films are summarized in Table 3. It can be observed that for the as-deposited films, the increase of the nitrogen content from 10 to 40% resulted in a shift of the Gaussian peaks’ position from 493 nm and 524 nm to 498 nm and 530 nm, respectively. This could be attributed to the increased defect generation and disordering of the ZnO lattice by nitrogen doping, as detected by the Raman measurements above. During heat treatment, these defects were partially annealed and, as a consequence, the Gaussian peaks moved to smaller wavelengths with increasing RTA temperature.

The above Raman and PL measurements show that inherent structural defects and N-induced defect centers were present in the ZnO films. These can be associated with localized states in the energy gap of ZnO, which are traps for electric charge carriers. For example, it has been established that the dominant defect centers of oxygen vacancy (PL peak at ~ 492 nm) develop deep energetic levels of 1.4 eV for electrons and 1.6 eV for holes in the ZnO bandgap [42].

### 3.3. Electric Charge Transport Properties

**Figure 3:**
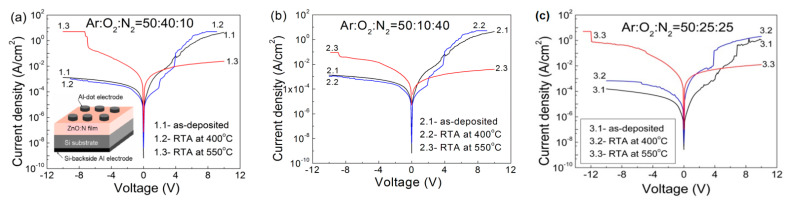
Automatically recorded I–V characteristics of MIS structures with ZnO:N films, deposited at different gas ratios: Ar:O_2_:N_2_ = 50:40:10 (**a**), Ar:O_2_:N_2_ = 50:10:40 (**b**), and Ar:O_2_:N_2_ = 50:25:25 (**c**). The corresponding sample numbers are given in the insets. The lower left inset in (**a**) shows the schematic structure of the measured samples.

In order to prove the role of deep levels in the conduction mechanism and to study the current via these deep levels, we conducted a detailed study of the I–V characteristics of the MIS structures formed with the ZnO:N films. The I–V characteristics of the MIS structures with the ZnO:N films, automatically recorded at room temperature, are summarized in Figure 3. A schematic representation of the studied MIS structures is shown as the lower left inset in Figure 3a.

In the case of as-deposited ZnO:N films (samples 1.1, 2.1, and 3.1) and those after RTA treatment at 400 °C (samples 1.2, 2.2 and 3.2), the current at positive voltages applied to the top Al-dot electrode was higher than the current in the case of negative voltage applied to the same contact. These MIS structures had diode-like properties. At absolute voltages of 8–10 V, the rectification ratios were ~4600, 7055, and 2500 for the as-deposited films obtained at 10%, 25%, and 40% N_2_ in the gas mixture, respectively. This ratio decreased after 400 °C RTA but still remained in the order of 10^3^. The strongest rectification effect was observed in ZnO:N films grown at 25% N_2_ in the gas mixture. The existence of asymmetry between the currents at positive and negative voltage values confirms the formation of n-ZnO–p-Si heterojunction. From these observations, it follows that the nitrogen-doped ZnO films were initially n-type and remained n-type after 400 °C annealing. In the case of the films deposited at gas ratios of Ar:O_2_:N_2_ = 50:40:10 and Ar:O_2_:N_2_ = 50:25:25, the comparison of the measured I–V curves of the as-deposited ZnO:N films with those of the corresponding ones after RTA at 400 °C showed close current values, which suggests similar concentrations of the N dopants.

For the samples after RTA at 550 °C (Figure 3, samples 1.3, 2.3, and 3.3), the current through the ZnO film was higher when negative voltage was applied to the top Al-dot electrode. The asymmetry between the currents at positive and negative voltage values was less pronounced, which was expressed in considerably smaller rectification ratios. At absolute voltages of 8–10 V, their values were 230, 534, and 24 for the films deposited at 10%, 25%, and 40% N_2_ in the gas mixture, respectively. These results imply that RTA at 550 °C transformed the nitrogen-doped ZnO films to p-type ZnO ones. In accordance with the Anderson heterojunction rule, the ZnO valence band offset at the p-ZnO–p-Si was close to 2.6 eV [17]. Because of this valence band offset, the contribution of holes in the p-Si to the forward current in the p-ZnO–p-Si MIS structure was negligible. Therefore, the forward current in these films was accompanied by electron-hole generation at the p-ZnO–p-Si interface.

It is known that acceptor dopants introduced into ZnO films are self-compensated by donor localized states generated in the ZnO lattice during the dopants’ incorporation in ZnO [20,43]. From the results in Figure 3, we can conclude that during RTA at 550 °C, the concentration of donor-like defects decreased in the ZnO:N films in such a way that the concentration of nitrogen-acceptor states exceeded that of the remaining donor-like defects. As a result, the n-type as-deposited ZnO:N film was transformed to a p-type one during 550 °C RTA. The I–V characteristics of the same MIS structures, measured after a year, showed only minor changes and still exhibited p-type conductivity in these ZnO:N films. Therefore, the transformation of n-type ZnO film into p-type by 550 °C RTA treatment is a stable process.

The transformation of ZnO:N film from n-type to p-type by 550 °C RTA is similar to the appearance of p-type regions in N-implanted ZnO single crystals after annealing at 500 °C, observed by deep-level transient spectroscopy (DLTS) measurements [44]. Similar transformations of N-implanted n-type ZnO films to p-type ones have been observed after RTA treatment at 900 °C [45].

The current densities in the ZnO:N films deposited with nitrogen contents of 10 and 25% in the vacuum chamber were similar (Figure 3). Deposition of ZnO:N films at the highest nitrogen content of 40% resulted in lower current density, and it remained lower in comparison to the other ZnO:N films even after RTA treatment at 550 °C. This result is a consequence of already mentioned self-compensation of acceptor dopants by the simultaneously created donor-like deep levels during the acceptor doping of ZnO.

In ZnO films doped with nitrogen, the n-type conduction can be attributed to donors formed by interstitial zinc, Zn_i_, and oxygen vacancies, V_O_, whereas the p-type conduction can be related to the acceptors formed by zinc vacancies, V_Zn_, oxygen interstitial, O_i_, and nitrogen substituting oxygen in the O sublattice [18,46,47]. We have confirmed the presence of these kinds of defect states in ZnO:N films by the analysis of IRSE and FTIR spectra [25] and also by the Raman and PL spectroscopic results presented and discussed above in Sections 3.1 and 3.2.

**Figure 4:**
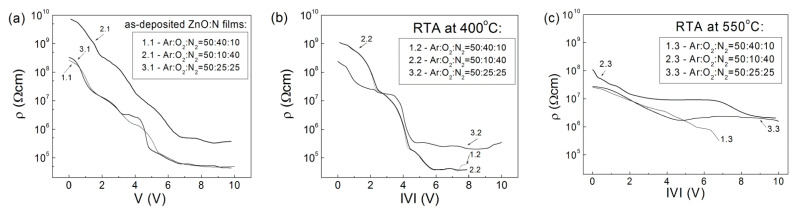
Specific resistivity ρ(V) versus applied forward voltage of ZnO:N films (deposited at different gas ratios (**a**) and after RTA at 400 °C (**b**) and 550 °C (**c**). The ρ values were calculated from the forward I–V curves shown in Figure 3.

The specific resistivity, ρ(V), values of the ZnO films were calculated from the I–V characteristics shown in Figure 3, in the forward direction with accumulation conditions at the ZnO–p-Si interface, using the relations R_dif_ = dV/dI and ρ(V) = R_dif_S/d_f_, where R_dif_ is the differential resistance, d_f_ is the film thickness, and S is the Al-dot contact area. The obtained ρ(V) values for the studied ZnO:N films are summarized in Figure 4.

For the as-deposited and 400 °C RTA n-ZnO:N films (Figure 4a,b), the specific resistivity decreased sharply by more than four orders of magnitude upon increasing the applied voltage from 1 to 8 V. After 550 °C RTA (Figure 4c), the ρ(V) values of the p-ZnO:N films decreased more gradually, as the reduction was approximately two orders of magnitude in the same voltage interval of 1–8 V, but as a whole, they remained above the range of 10^6^ Ω cm. In comparison with the as-deposited ZnO:N films, the smaller change of the specific resistivity in the 550 °C RTA-treated films as a function of the applied voltage is connected with the smaller increase of the current density in the 550 °C RTA films.

In order to elucidate the role of deep levels in the ZnO:N energy gap, we further measured manually, pointbypoint, the I–V characteristics of the MIS structures with ZnO:N films. For these experiments, we chose the sample series 2.1–2.3 with maximal concentration of nitrogen in the ZnO film, expecting a larger effect of N doping on the electrical characteristics.

**Figure 5:**
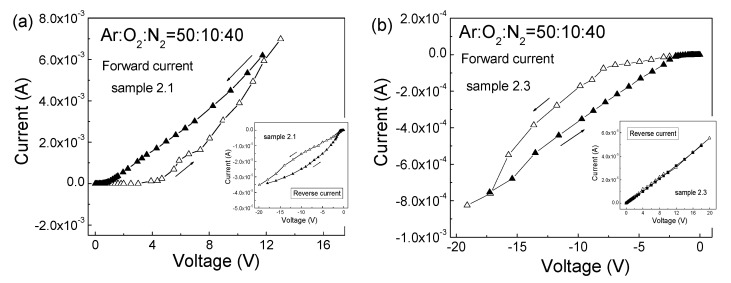
Room temperature I–V characteristics of MIS structures with ZnO:N films, deposited at Ar:O_2_:N_2_ = 50:10:40 (sample 2.1) (**a**) and after RTA at 550 °C (sample 2.3) (**b**). The initial and return stages of the I–V measurements are denoted with empty and full triangles, respectively. The corresponding I–V curves in the reverse direction are given as insets.

The room-temperature I–V characteristics of the MIS structure with ZnO:N films deposited at Ar:O_2_:N_2_ = 50:10:40 (sample 2.1) (a) and after RTA at 550 °C (sample 2.3) (b) are presented in Figure 5. To reveal the participation of deep levels in the ZnO energy gap, two-stage I–V measurements were performed. For the as-deposited n-type ZnO films of sample 2.1 (Figure 5a), the initial stage was measured in 20–25min increments starting from zero up to the maximal applied voltage, V_a_, and the second, return stage was carried out immediately after the initial one, down from V_a_ to zero also in 20–25min increments. The corresponding I–V curves in the reverse direction are given as an inset in Figure 5a. The considerable asymmetry between the currents at positive and negative voltage values confirmed the formation of the n-ZnO:N–p-Si heterojunction. The forward current in the return stage was higher than the forward current in the initial stage, resulting in a counterclockwise hysteresis in the measured I–V curves. Some of the electrons injected into the ZnO film during the initial stage were captured at deep levels with time constants higher than the time needed for measuring the initial stage, and their release during the return stage led to the observed excess, higher forward current in the return stage. Thus, the hysteresis in the I–V characteristics indicates that deep levels in the ZnO energy gap take part in the charge transport mechanism through the n-ZnO:N films.

The forward I–V characteristics of the MIS structures with ZnO:N films annealed at 550 °C (sample 2.3) are shown in Figure 5b, withthe corresponding I–V curves in reverse direction are given as inset. As expected, the forward current was with negative voltages applied to the Al-dot contact on the ZnO:N surface. As shown in Figure 5b, the forward current in the return stage was also higher than the forward current in the initial stage, leading to the appearance of counterclockwise hysteresis in the forward I–V characteristics. This counterclockwise hysteresis shown in Figure 5b, as the one in Figure 5a, also confirms that the deep levels in the ZnO energy gap take part in the charge transport mechanism in the p-ZnO:N films.

**Figure 6:**
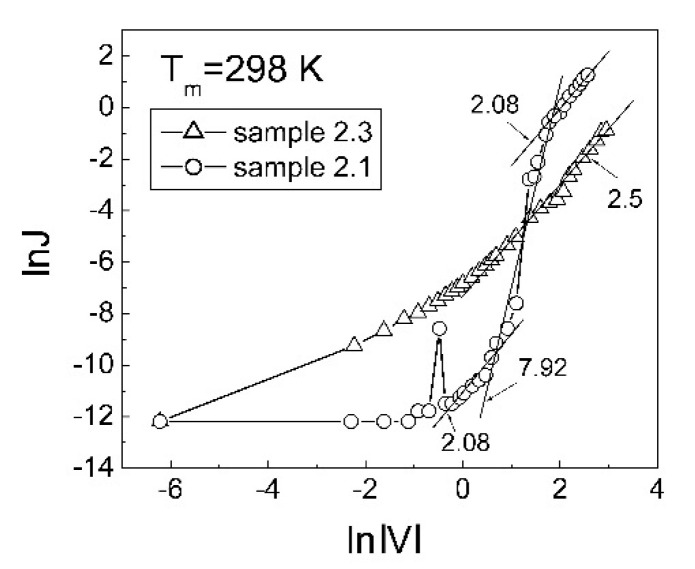
Logarithm of room-temperature forward current density as a function of logarithm absolute voltage for the MIS structures with ZnO:N films, deposited at Ar:O_2_:N_2_ = 50:10:40 (sample 2.1) and after RTA at 550 °C (sample 2.3).

The room-temperature dependences of ln(J) versus ln|V| in the forward direction and in the initial stage are presented in Figure 6. For the MIS structures with as-deposited ZnO:N films, the slope of the plots is about 8 in the dominant parts of the plots and, around 2 for the other parts. This means that the charge transport in these ZnO:N films is carried out via deep levels in the ZnO energy gap. This transport mechanism is described as trap charge limited current (TCLC) (see for example [48]) or trap-assisted space charge limited current (see for example [49]). For the MIS structures with 550 °CRT-treated ZnO:N films, the slope of the lnJ versus ln|V| plot (Figure 6) is around 2.5 in the range of 2–18 V. Since this slope is also higher than 2, this means that the charge transport in the p-type ZnO:N is also carried out via deep levels in the ZnO energy gap.

**Figure 7:**
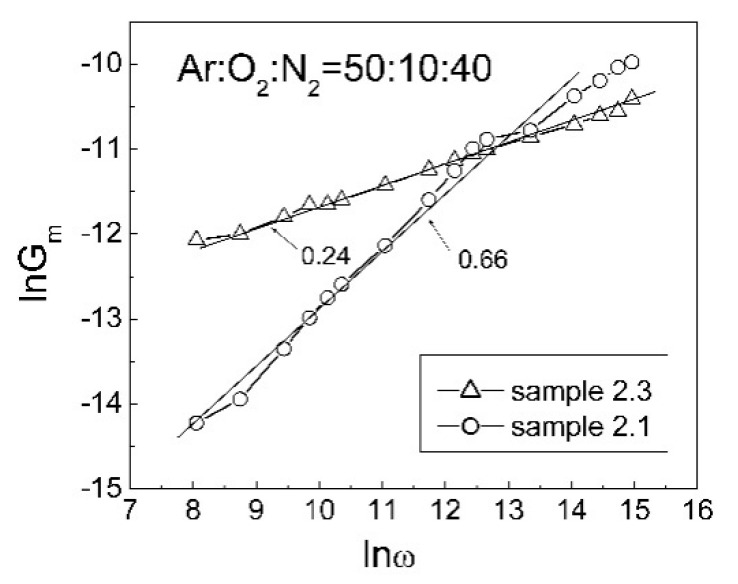
Logarithm of AC conductance, G_m_, versus logarithm of angular frequency, ω, of MIS structures with ZnO:N films deposited at Ar:O_2_:N_2_ = 50:10:40 (sample 2.1) and after RTA at 550 °C (sample 2.3).

The AC conductance, G_m_, of these ZnO:N films, measured in the 0.5–500 kHz test frequency range, is plotted in Figure 7 as lnG_m_ against lnω, where ω is the angular frequency. The slope of the plot for the as-deposited n-ZnO:N film (sample 2.1) is equal to 0.66. This value is close to the most widespread value 0.7 of the power exponent in the Jonscher universal power law for AC conductance in the case of hopping or tunneling of charge carriers via deep levels near to the Fermi level in the semiconductor energy gap [50]. For the annealed p-ZnO:N films (sample 2.3), the slope of this plot is equal to 0.24.

**Figure 8:**
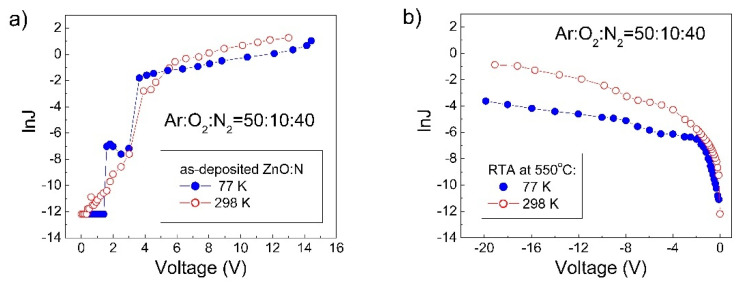
lnJ versus V characteristics, measured at 298 and 77 K, of MIS structures with ZnO:N films deposited at Ar:O_2_:N_2_ = 50:10:40 (sample 2.1) (**a**) and after RTA at 550 °C (sample 2.3) (**b**).

In order to reveal the character of the charge transport mechanism through the ZnO:N film, current–voltage measurements must be done at different temperatures. In Figure 8a, the I–V characteristics of the MIS structure with as-deposited n-ZnO:N, measured at 298 and 77 K, are given as the logarithm of the forward current density versus applied voltage. The averaged current density measured at 298 K under high applied voltages was 2.53 times higher than the current density measured at 77 K. Using the expression ln(J_298_/J_77_) = (qφ_a_/k) (1/77–1/298), the effective activation energy, qφ_a_, of these current densities was estimated. For the as-deposited n-ZnO:N film, it was equal to qφ_a_ = 8.3 meV. This small qφ_a_ value indicates that the current in this film was carried out predominantly by electron tunneling from the occupied deep level to the nearest unoccupied one in the ZnO energy gap. The current at 77 K was carried out by intertrap tunneling mechanism [43]. The measured excess current at 298 K is a consequence of the appearance of thermally activated carrier hopping in these ZnO:N films at temperatures higher than 77 K [51].

In the case of intertrap tunneling, the current density J is given by

J = J_0_ sinh[B(V − V_fb_)] (1)

where J_0_ = 2qν{exp[−2(2m*q)^1/2^φ_t_^1/2^w/ħ]}/w^2^ and B = (2m*q)^1/2^w^2^/ħφ_t_^1/2^d_f_ [52]; w is the mean distance between deep levels situated around the quasi-Fermi level; ν is the electron attempt to escape frequency from deep levels; and qφ_t_ is the energy position of deep levels in the ZnO energy gap. The electron effective mass, m_n_* for n-type ZnO is taken as equal to 0.23 m_e_ [53]. The electron attempts to escape frequency, ν = 8.54 × 10^12^ s^−1^, is estimated from the relation hν = kT_D_ [54], where the Debye temperature, T_D_, for wurtzite ZnO is 410 K [55], and all other symbols have their common meaning. From Equation (1) it follows that the values of w and qφ_t_ can be determined from the slope of the plot lnJ versus V and the intersection of the extension of this plot toward the lnJ axis at V = 0. These values of w and qφ_t_ determined from the lnJ-V plot in Figure 8a at 77 K were4.71 × 10^−7^ cm and 1.48 eV, respectively. The density of deep levels N_t_ in the ZnO energy gap was estimated by the expression N_t_ = 1/w^3^ and it was equal to N_t_ = 9.62 × 10^18^ cm^−3^. Because of the energy position of deep levels is qφ_t_ = 1.48 eV, the position of electron quasi-Fermi level in these n-ZnO MIS structures was 0.12 eV above the middle of the ZnO energy gap.

When the concentration of deep levels is known, one may calculate the mobility μ_t_ for this intertrap tunneling charge transport by the relation μ_t_ = J/(qN_t_E), where J is the current density at the applied voltage V and E is the electric field across the ZnO film. In the accumulation conditions at the ZnO/Si interface, E is given by V/d_f_, where d_f_ is the ZnO film thickness. The calculated low value of μ_t_ = 1.04 × 10^−6^ cm^2^V^−1^s^−1^ is characteristic for charge transport via deep levels in the semiconductor band gap.

The excess of the current at 298 K over that at 77 K (Figure 8a) is evidence for thermally activated carrier hopping in these n-ZnO MIS structures. N.F. Mott [51] has proposed that in the case of variable range hopping (VRH), the temperature-dependent conductivity can be expressed by σ(T) = σ_0_exp{−(T_0_/T)^1/4^}, where σ is the specific conductivity and T_0_ is the characteristic temperature. This dependence is appropriate for the Mott VRH in ZnO [56,57,58]. One may calculate the value of characteristic temperature T_0_ by using the expression ln(J_298_/J_77_) = T_0_^1/4^[1/(77)^1/4^ − 1/(298)^1/4^], where the current density is averaged over high applied voltages. For the as-deposited n-ZnO MIS structures (Figure 8a) this value was T_0_ = 8.45 × 10^3^ K.

The localization length, Bohr radius, a_B,ZnO_, of the localized states in the ZnO energy gap is given by the expression a_B,ZnO_ = (4πεZnOħ^2^)/(m*q^2^), where ε_ZnO_ is the dielectric constant of ZnO, m* is the effective electron mass in ZnO, and other symbols have their usual meanings. In the corresponding 1 MHz C-V curve of sample 2.1 (not shown in this paper), the measured maximal capacitance value in the accumulation regime was equal to C_max_ = 98 pF. As C_max_ is expressed by C_max_ = εε_0_S/d_f_, the dielectric constant ε_ZnO_ of the ZnO:N film was determined as ε_ZnO_ = εε_0_ = 1.03 × 10^−12^ F/cm. Knowing ε_ZnO_ and m_n_* = 0.23 m_e_ [53] quantities, the calculated value of a_B,ZnO_ was equal to a_B,ZnO_ = 2.674 × 10^−7^ cm.

The density of localized states, N(ε), in the case of hopping mechanism is given by the expression N(ε) = 16α^3^/kT_0_, where the decay constant α is the inverse of the Bohr radius (α = 1/a_B,ZnO_). The numerical factor 16 is taken from Ambegaokar et al. [59]. With α = 3.74 × 10^6^ cm^−1^ and T_0_ = 8.449 × 10^3^ K the calculated value of N(ε) was equal to 1.15 × 10^21^ cm^−3^.eV^−1^. When N(ε) is known, the most probable hopping distance R_m_ can be estimated by the expression R_m_ = (9/8παN(ε)kT)^1/4^ [60,61]. At the temperature of 187.5 K, taken as the middle value of temperature range of 77–298 K, R_m_ was calculated, and it was equal to R_m_ = 2.677 × 10^−7^ cm. This R_m_ value is practically equal to the localization radius a_B,ZnO_ = 2.67 × 10^−7^ cm, and it is 14 % higher than the half-distance w = 2.35 × 10^−7^ cm between adjacent deep levels obtained from the I–V measurement at 77 K. The most probable energy difference of deep levels, ∆W, taking part in the electron hopping is given by ∆W = (3/4πR_m_^3^N(ε)) [60,61]. The calculated value of ∆W = 10.78 meV for this hopping in the n-ZnO–p-Si MIS structures was close to the effective thermally activation energy qφ_a_ = 8.3 meV in the as-deposited n-ZnO films. Therefore, in addition to temperature-independent intertrap tunneling, thermally activated hopping also took place in these n-type ZnO films in the temperature range of 77–298 K.

In Figure 8b, the forward I–V characteristics, measured at 298 and 77 K, of the MIS structures with ZnO:N films after RTA at 550 °C (sample 2.3) are given as the logarithm of the current density versus applied voltage. The averaged current density measured at 298 K under high applied voltage was 17.21 times higher than the current density measured at 77 K. The effective activation energy of the current densityof this p-ZnO:N film, obtained from the expression ln(J_298_/J_77_) = (qφ_a_/k)(1/77 − 1/298) was equal to qφ_a_ = 25.46 meV. This value shows that the current in these p-ZnO:N films was also carried out predominantly by tunneling of holes from the occupied deep level to the nearest unoccupied one in the ZnO:N energy gap. Because of that, at 77 K the current density J is also given by the equation (1), where the electron effective mass, m_n_* = 0.23 m_e_, is replaced with the hole effective mass, m_h_* = 0.59 m_e_ [62].

The values of qφ_t_ and w, calculated from the slope of the plot lnJ versus V in Figure 8b and the intersection of the extension of this plot toward lnJ axis at V = 0, were qφ_t_ = 1.27 eV and w = 3.724 × 10^−7^ cm, respectively. Under high forward bias the concentration of injected holes in the annealed at 550 °C p-ZnO film was equal to N_t_ = 1/w^3^ = 1.94 × 10^19^ cm^−3^. Taking into account that our previous EDX studies have shown a nitrogen/oxygen ratio of 1/8 in both RF-sputtered and annealed ZnO:N films [25], the total concentration of nitrogen in the 550 °C RTA p-ZnO film (sample 2.3) was 1.82 × 10^21^ cm^−3^. The comparison between the concentration of acceptor deep levels (N_t_ = 1.94 × 10^19^ cm^−3^) with the total concentration of N atoms incorporated into ZnO:N films (1.82 × 10^21^ cm^−3^) shows that less than 1% of acceptor levels were not compensated. Because of this strong charge compensation, it is difficult to achieve an increase in the conductivity of ZnO by introducing a larger amount of nitrogen into a ZnO:N film.

Assuming that temperature-dependent current in these p-type ZnO:N films is also governed by the Mott VRH [51], the characteristic temperature T_0_ obtained from the relation ln(J_298_/J_77_) = T_0_^1/4^[1/(77)^1/4^ − 1/(298)^1/4^] was equal to T_0_ = 7.44 × 10^5^ K. The values of the Bohr radius a_B,ZnO_, the decay constant α, the density of localized states, N(ε), the most probable hopping distance, R_m_, and the most probable hopping energy difference, ∆W, were calculated in the same way as the corresponding parameters for the n-type ZnO:N films. The obtained values were, respectively, a_B,ZnO_ = 1.042 × 10^−7^ cm, α = 9.59 × 10^6^ cm^−1^, and N(ε) = 2.2 × 10^20^ cm^−3^.eV^−1^, and at 187.5 K the R_m_ and ∆W values were3.2 × 10^−7^ cm and 33.07 meV, respectively. These results reveal that in the p-type ZnO films, the most probable hopping distance R_m_ was about three times larger than the Bohr radius a_B,ZnO_ and this relation confirms that Mott variable range hopping of holes occurred in these p-type ZnO:N films. Therefore, the additional current at 298 K compared to that at 77 K in these p-type ZnO:N films was carried out by charge carrier hopping, close to the Fermi or the quasi-Fermi level in the ZnO:N energy gap.

For better insight, the above calculated charge transport parameters of the intertrap tunneling and variable range hopping mechanism in ZnO:N films deposited at a gas ratio of Ar:O_2_:N_2_ = 50:10:40 (sample 2.1) and treated by RTA at 550 °C (sample 2.1) are summarized in Table 4.

The decrease of the ZnO band gap density of localized states from N(ε) = 1.15 × 10^21^ cm^−3^eV^−1^ for the as-deposited ZnO:N (sample 2.1) to N(ε) = 2.2 × 10^20^ cm^−3^eV^−1^ for the p-ZnO:N after 550 °C RTA (sample 2.3) correlates well with the observed decrease in the slope values of lnG_m_ versus lnω plots (Figure 7) from 0.66 to 0.24, respectively. The decrease of the density of localized states in the ZnO bandgap by the 550 °C RTA is evidence that the concentration of donor-like defects decreased during this treatment. The densities of localized states N(ε) given in Table 4 are within the range of 9.93 × 10^19^–2.08 × 10^22^ cm^−3^eV^−1^ reported for VRH conduction in n-type polycrystalline ZnO films [63]. Values of N(ε) = 2×10^20^ cm^−3^eV^−1^ and Δ*W* = 29 meV, close to ours, for the densities of localized states and corresponding characteristic energies, respectively, have been obtained from the current–voltage characteristics of amorphous ZnON thin film transistors [64].

The established transformation of n-type conduction to p-type in the studied ZnO:N films viaannealing the samples at 550 °C suggests that if the deposition could be accomplished at such elevated temperatures as 550 °C, the density of localized states could be reduced below N(ε) = 2.2 × 10^20^ cm^−3^.eV^−1^, as was obtained in the 550 °C RTA-treated ZnO:N films. In this way, it would be possible to obtain nitrogen-doped ZnO films with weaker N-acceptor self-compensation and a smaller density of localized states in the p-type ZnO band gap than that we observed herein.

## 4. Conclusions

Nitrogen-doped ZnO thin films were deposited onto Si substrates by RF magnetron sputtering in nitrogen-containing ambient gas, followed by rapid thermal annealing at 400 °C and 550 °C in nitrogen. Raman and PL spectroscopic analyses revealed defects due to O and Zn vacancies and interstitials, which are related to carrier traps in the ZnO:N films.

Detailed analyses of the I–V characteristics of the MIS structures with the ZnO:N films revealedn-type conduction in both as-deposited and annealed at 400 °C ZnO:N films. The reason for n-type conductivity in these ZnO:N films is a result of the self-compensation of the N-acceptor levels by donor-like defects generated during N doping. RTA at 550 °C decrease the concentration of donor-like defects below the concentration of N acceptors and, as a consequence, the n-type ZnO:N films transformed into p-type ZnO:N ones. Repeated measurements after a year proved that the resulting conduction-type transformation at the annealing stage at 550 °C is a stable process.

The observed counterclockwise hysteresis in the measured point-by-point I–V characteristics of both n- and p type ZnO:N films confirms the presence of deep levels in the ZnO energy gap which participate in the charge transport through these ZnO:N films. The slope of the lnJ versus lnV plots reveals that the current in both n- and p-type ZnO:N films is trap charge limited current (TCLC), while the slope of the lnG_m_ versus lnω plots confirms the tunneling or hopping of charge carriers via deep levels in the ZnO energy gap.

At 77 K, the current in both n- and p-type ZnO:N films is carried out by intertrap tunneling via deep levels in the ZnO energy gap. The higher forward averaged current density at 298 K than that measured at 77 K reveals additional thermally activated variable range carrier hopping in these ZnO:N films.

## Figures and Tables

**Table 1:** Technological conditions and corresponding sample number of ZnO:N films with the given layer’s thickness.

ZnO:N Sample Number	Gas Mixture of Ar:O_2_:N_2_(%)	RTA Temperature (°C)	ZnO:N Film Thickness (nm)
1.1	50:40:10	as-deposited	214.8
1.2		400	183.8
1.3		550	185.7
2.1	50:10:40	as-deposited	206.6
2.2		400	180.7
2.3		550	202.2
3.1	50:25:25	as-deposited	106.4
3.2		400	127.0
3.3		550	124.4

**Table 2:** Position of the Raman phonon modes in the ZnO:N thin films.

Gas Ratio of Ar:O_2_:N_2_ (%)	RTA Temperature (°C)	Raman Molecular Vibration Modes (cm^−1^)					
50:40:10	as-deposited	221	332	373	411	555	589
	400	224	338	377	415	555	590
	550	234	334	378	409	557	595
50:25:25	as-deposited	237	336	376	412	553	594
	400	233	329	372	402	552	590
	550	236	330	376	418	551	593
50:10:40	as-deposited	236	330	376	402	552	593
	400	238	335	375	407	554	591
	550	239	337	376	411	550	595

**Table 3:** The positions of the PL Gaussian emission peaks of the ZnO:N films.

Gas Ratio of Ar:O_2_:N_2_ (%)	RTA Temperature (°C)	Blue Green Spectral Range (nm)	Green Spectral Range (nm)
50:40:10	as-deposited	493	524
	400	497	522
	550	497	525
50:25:25	as-deposited	495	527
	400	495	523
	550	495	522
50:10:40	as-deposited	498	530
	400	496	525
	550	490	524

**Table 4:** Intertrap tunneling and variable range hopping parameters of ZnO:N films deposited at gas ratio of Ar:O_2_:N_2_ = 50:10:40 (sample 2.1) and treated by RTA at 550 °C (sample 2.1): qφ_a_—effective thermally activation energy; w—mean distance between deep levels; qφ_t_ and N_t_—energy position and density of deep levels, respectively, in the ZnO energy gap; T_0_—characteristic temperature; a_B,ZnO_—Bohr radius of the localized states in the ZnO energy gap; N(ε)—density of localized states; R_m_ and ∆W—most probable hopping distance and energy difference of deep levels, respectively.

Parameters	As-Deposited ZnO:N	After RTA at 550 °C
**Intertrap tunneling**		
qφ_a_ (meV)	8.3	25.46
w (cm) at 77 K	4.70 × 10^−7^	3.724 × 10^−7^
qφ_t_(eV) at 77 K	1.48	1.27
N_t_(cm^−3^) at 77 K	9.62 × 10^18^	1.94 × 10^19^
**Variable range hopping**		
T_0_(K)	8.45 × 10^3^	7.44 × 10^5^
a_B_,_ZnO_(cm)	2.67 × 10^−7^	1.04 × 10^−7^
N(ε)(cm^−3^.eV^−1^)	1.15 × 10^21^	2.2 × 10^20^
R_m_ (cm) at 187.5 K	2.677 × 10^−7^	3.2 × 10^−7^
∆W (meV) at 187.5 K	10.78	33.07

## Data Availability

The data presented in this study are available on request from the corresponding author.
